# Quantitative proteomic analysis of SARS-CoV-2 infection of primary human airway ciliated cells and lung epithelial cells demonstrates the effectiveness of SARS-CoV-2 innate immune evasion

**DOI:** 10.12688/wellcomeopenres.17946.1

**Published:** 2022-09-07

**Authors:** Thomas W.M. Crozier, Edward J.D. Greenwood, James C. Williamson, Wenrui Guo, Linsey M. Porter, Ildar Gabaev, Ana Teixeira-Silva, Guinevere L. Grice, Arthur Wickenhagen, Richard J. Stanton, Eddie C. Y. Wang, Sam J. Wilson, Nicholas J. Matheson, James A. Nathan, Frank McCaughan, Paul J. Lehner

**Affiliations:** 1 Department of Medicine, Cambridge Institute of Therapeutic Immunology and Infectious Disease, University of Cambridge, Cambridge, CB2 0AW, UK; 2 Department of Medicine, Addenbrookes Hospital, University of Cambridge, Cambridge, CB2 0QQ, UK; 3 MRC - University of Glasgow Centre for Virus Research, Glasgow, G61 1QH, UK; 4 Division of Infection and Immunity, School of Medicine, Cardiff University, Cardiff, CF14 4XN, UK; 5 NHS Blood and Transplant, Cambridge, CB2 0PT, UK

**Keywords:** SARS-CoV-2, COVID-19, Coronavirus, proteomics

## Abstract

**Background: **Quantitative proteomics is able to provide a comprehensive, unbiased description of changes to cells caused by viral infection, but interpretation may be complicated by differential changes in infected and uninfected ‘bystander’ cells, or the use of non-physiological cellular models.

**Methods: **In this paper, we use fluorescence-activated cell sorting (FACS) and quantitative proteomics to analyse cell-autonomous changes caused by authentic SARS-CoV-2 infection of respiratory epithelial cells, the main target of viral infection *in vivo*. First, we determine the relative abundance of proteins in primary human airway epithelial cells differentiated at the air-liquid interface (basal, secretory and ciliated cells). Next, we specifically characterise changes caused by SARS-CoV-2 infection of ciliated cells. Finally, we compare temporal proteomic changes in infected and uninfected ‘bystander’ Calu-3 lung epithelial cells and compare infection with B.29 and B.1.1.7 (Alpha) variants.

**Results: **Amongst 5,709 quantified proteins in primary human airway ciliated cells, the abundance of 226 changed significantly in the presence of SARS-CoV-2 infection (q <0.05 and >1.5-fold). Notably, viral replication proceeded without inducing a type-I interferon response. Amongst 6,996 quantified proteins in Calu-3 cells, the abundance of 645 proteins changed significantly in the presence of SARS-CoV-2 infection (q < 0.05 and > 1.5-fold). In contrast to the primary cell model, a clear type I interferon (IFN) response was observed. Nonetheless, induction of IFN-inducible proteins was markedly attenuated in infected cells, compared with uninfected ‘bystander’ cells. Infection with B.29 and B.1.1.7 (Alpha) variants gave similar results.

**Conclusions: **Taken together, our data provide a detailed proteomic map of changes in SARS-CoV-2-infected respiratory epithelial cells in two widely used, physiologically relevant models of infection. As well as identifying dysregulated cellular proteins and processes, the effectiveness of strategies employed by SARS-CoV-2 to avoid the type I IFN response is illustrated in both models.

## Introduction

SARS-CoV-2 will continue to circulate in the human population. Efforts to minimise the loss of life and damage to healthcare systems caused by COVID-19 will rely on vaccines and the further development of anti-viral therapies to help prevent disease in infected patients. Intense research on SARS-CoV-2 will therefore continue into the foreseeable future.

A clear understanding of the pathways and mechanisms exploited by SARS-CoV-2 in the host cell is an important foundation of SARS-CoV-2 research. This can best be achieved using techniques which map these changes globally, and in an unbiased fashion. Primary human airway epithelial cells (hAECs) grown at the air-liquid interface (ALI) represents one of the most compelling primary cell models for SARS-CoV-2 infection. At the ALI, hAECs differentiate into a pseudostratified epithelium consisting of mixed cell types of the human airway, which show varying susceptibility to SARS-CoV-2 infection ^1– 3^. Previous studies have characterised the transcriptional changes to ALI-AECs following SARS-CoV-2 infection, using either bulk RNA-seq on a mixture of infected or uninfected cells, or using scRNA-seq ^2, 4– 7^. However, relying on transcriptional changes to infer the SARS-CoV-2 effect on host cell proteins is complicated, as SARS-CoV-2 infection induces a host translational shutoff which disrupts the connection between transcription and protein abundance ^8^. Further, viruses frequently act to disrupt proteins and protein complexes directly rather than through altering transcription, for example, by targeting proteins for degradation ^9– 14^.

Here, we used Tandem Mass Tag (TMT)-based quantitative proteomics to characterise the proteomic landscape of the primary cells found in the human airway epithelium. We focussed on the ciliated cells as SARS-CoV-2 infection of ALI-AEC’s revealed this cell type to be most permissive to infection. Combining formaldehyde fixation and permeabilisation with immunostaining for SARS-CoV-2 nucleocapsid protein and fluorescence-activated cell sorting (FACS) allowed us to separate pure populations of infected ciliated cells from the uninfected (‘bystander’) populations. We characterised the changes to the host cell proteome caused by infection in each of these populations. Technical limitations of using these cells limited our proteome coverage, so we applied the same methodology to extend our observations to pure populations of infected and uninfected cells of the human epithelial cell line Calu-3.

## Methods

### Cell culture

hAECs were purchased from Lonza (Cat. no. # CC-2540, male). Primary airway cells at passage 2 were expanded in PneumaCult-Ex Plus Medium (Cat. no. #05040; Stemcell) then seeded on collagen (Cat. no. #354236; Corning) coated 24-well Transwell inserts with 0.4-μm pores (Cat. no. #353095; Falcon) until fully confluent. After reaching confluency, cells were taken to the ALI and maintained in PneumaCult-ALI Medium (Cat. no. #05021; Stemcell) for ≥28 days prior to infection with SARS-CoV-2.

Calu-3 cells (ATCC HTB-55) were obtained from a collaborator as detailed in the acknowledgments. They were maintained in Minimum Essential Media (MEM) with 10% fetal calf serum (FCS), GlutaMAX, 1 mM sodium pyruvate and MEM non-essential amino acids (NEAA). ACE2 high Calu-3 single cell clones were generated by plating at a limiting dilution into 96-well plates. Clones were screened for the ability to bind full length biotinylated SARS-CoV-2 spike protein tetramerised via streptavidin-AF647 by flow cytometry, as described previously ^15^

### SARS-CoV-2 infections

The SARS-CoV-2 viruses used in this study were the clinical isolates named “SARS-CoV-2/human/Liverpool/REMRQ0001/2020” ^16, 17^ (Lineage B.29) and “SARS-CoV-2 England/ATACCC 174/2020” (Lineage B.1.1.7) ^17, 18^. Viral stocks were obtained from collaborators as detailed in the acknowledgments. Viral stocks were sequenced prior to use and the consensus sequence matched the expected sequence exactly. For sequencing of viral stocks, Calu-3 cells cultured in 24-well plates were infected with viral supernatant at a low (<0.1) multiplicity of infection (MOI). Total cellular RNA was harvested 48–72 h post-infection utilising RNeasy Plus Mini Kit (Qiagen, 74134) following manufacturer recommended protocol for samples <5 × 10 ^6^ cells. Extracted RNA was sequenced as described previously ^19^ using a MinION instrument (Oxford Nanopore Technologies, Oxford, UK) and following ARTICNetwork V3 protocol ( https://dx.doi.org/10.17504/protocols.io.bbmuik6w), with sequence assembly using ARTICNetwork assembly pipeline. Viral titre was calculated by 50 % tissue culture infectious dose (TCID _50_) in Huh7-ACE2 cells.

For viral infection of hAECs at ALI, 50 μL of viral containing supernatant was added to the apical side of transwells for 2–3 h, then removed. At 72 h post-infection hAEC-ALI transwells were harvested for flow cytometry analysis or FACS as described below.

For viral infection of Calu-3, cells were plated 72 h prior to addition of SARS-CoV-2 virus. One well was used for cell counting to calculate the viral dose required to achieve the indicated MOI for each experiment. Cells were harvested as described below at 8, 24 or 48 h post infection.

### Flow cytometry and cell sorting

For analysis of hAEC-ALI cell-type specific markers, trans-wells cultured for 22 days at ALI were washed once with phosphate-buffered saline (PBS) and incubated in TrypLE at 37°C for 15 min. Cells were dissociated with gentle pipetting and neutralised in DMEM + 10% FCS and pelleted by centrifugation (Eppendorf 5810 R) at 400 g for 5 min, resuspended in 0.5% formaldehyde in PBS and incubated for 15 min. Cell pellets were washed three times in cold FCS-PBS (5% FCS in PBS) then cell-surface stained for 30 min on ice with PE-conjugated anti-NGFR (clone ME20.4, mouse monoclonal, Biolegend 345105, 1:1000 dilution) and BV605-conjugated anti-CEACAM6 (clone B6.2, mouse monoclonal, BD 742685, 5 μg/mL) antibodies diluted in FCS-PBS. Cells were washed, permeabilised in 0.2% saponin in FCS-PBS for 15 min on ice, then stained with AF647-conjugated anti-acetylated α-tubulin (clone 6-11B-1, Santa Cruz sc-23950 AF647, 0.02 μg/mL) antibody (AF647-TUBA) for 40 min prior to washes in FCS-PBS and either flow cytometry analysis or FACS. Cells were sorted into four populations: AF647-TUBA+; PE-NGFR+; BV605-CEACAM6+ and unstained cells. A minimum of 80,000 cells were collected for each cell type and pelleted for proteomic analysis.

SARS-CoV-2 infected hAEC-ALI trans-wells were prepared similarly with a few modifications. Cells were detached in TrypLE at room temperature for 20–30 min and added directly to PBS containing formaldehyde to a final concentration of 4% for 15 min for flow cytometry analysis or 2% for 30 min for FACS-proteomic analysis. Cells were permeabilised and stained for 15 min at room temperature with AF647-TUBA and sheep anti-SARS-CoV-2 nucleoprotein ^20^ (sheep polyclonal, MRC-PPU DA114, 0.7 μg/mL) antibodies, washed, and incubated with AF488 donkey anti-sheep antibody (#713-545-147; Jackson ImmunoResearch; 2 μg/mL) for 15 min at room temperature. Ciliated cells (AF647-TUBA+) were sorted into SARS-CoV-2 nucleoprotein positive (infected) and negative (bystander/not-infected) populations, collecting a minimum of 27,000 cells for each condition tested for proteomic analysis.

SARS-CoV-2 infected Calu-3 cells were detached, fixed, permeabilised and stained as described for hAEC-ALI transwells, omitting AF647-TUBA antibodies. Calu-3 were sorted into SARS-CoV-2 nucleoprotein positive and negative populations, collecting a minimum of 160,000 cells for each condition tested for proteomic analysis.

### Whole cell proteomics with S-trap method

Whole cell proteomics was carried out as described previously ^21, 22^, with some modifications.

### Materials

All chemicals used were of analytical reagent grade or better and sourced from Sigma/Merck unless stated otherwise.

### Lysis and protein quantification

Cells pellets were resuspended in 76 mM HEPES pH 7.55, 3 mM MgCl _2_, Benzonase (1400 u/mL) and 15 mM TCEP. 20% SDS was then immediately added to the cell suspension (final 5%) using a low retention pipette tip (RPT, StarLab) and mixed by pipetting. Samples were incubated for 15 min at 55°C to complete reduction. Samples were then alkylated by adding MMTS to a final concentration of 15 mM, and incubating at RT for 15 min. For experiments with limited cell numbers, entire lysates were taken forward to digestion without quantification. Conversely, where a large quantity of cells were available 5 µL aliquots were taken, diluted 2x in water and quantified by reducing agent compatible BCA assay (Thermo Fisher). The standard curve consisted of 2000, 1500, 1000, 750, 500, 250, 125 and 25 µg/mL BSA in 2x diluted lysis buffer. 9 µL of samples or standards were mixed with 4 µL reconstituted reducing agent compatibility reagent and incubated at 37 degrees for 15 min. Subsequently 240 µL BCA reagent (50:1 Reagent A:B) was added and incubated at 37 degrees for a further 30 min. 200 µL of each standard/sample was transferred to a 96-well plate and absorbance read at 595nm in a plate reader (Clariostar, BMG labtech). A 2-order polynomial curve was fit to the standard curve and protein concentrations of samples derived from the equation. 25 µg of each sample was taken and the volumes of each lysate equalised using resuspension buffer +5% SDS.

### S-trap digestion

A 10% volume of 12% phosphoric acid was added to each sample to acidify samples to ~pH2, completing denaturation. 6x volumes of wash buffer (100 mM HEPES pH 7.1, 90% Methanol) was added and the solution was then loaded onto a µS-trap (Protifi) using a positive pressure manifold ((PPM), Tecan M10), not more than 150 µL of sample at a time (~100 PSI). Adaptors fabricated in-house were used to allow the use of S-traps with the manifold. Samples were then washed 4x with 150 µL wash buffer. To eliminate remaining wash buffer S-traps were centrifuged at 4000 g for 1 min. To each S-trap, 30 µL of digestion solution (50 mM HEPES pH 8, 0.1% Sodium Deoxycholate) containing 1.25 µg Trypsin/lysC mix (Promega) was added. S-traps were capped loosely and placed in low adhesion 1.5 mL microfuge tubes in a ThermoMixer C (Eppendorf) with a heated lid and incubated for 6 h at 37°C. Peptides were then recovered by adding 40 µL digestion buffer to each trap and then incubating at RT for 15 min before slowly eluting with positive pressure (2–3 PSI). Traps were then subsequently eluted with 40 µL 0.2% formic acid and then 40 µL 0.2% formic acid, 50% acetonitrile. Eluted samples were then dried in a vacuum centrifuge (Thermo Fisher, centrifuge (SC210A), cold trap (RVT5105), and vacuum pump (RV5 A65313906)).

### TMT labelling and clean-up

Samples were resuspended in 21 µL 100 mM TEAB pH 8.5. After equilibrating to room temperature, TMT reagents (Thermo Fisher) were resuspended in 9 µL anhydrous acetonitrile, which was then added to the respective samples and incubated at RT for 1 h. A 3 µL aliquot of each sample was taken and pooled to analyse TMT labelling efficiency and equality of loading by LC-MS. Samples were stored at -80°C in the intervening period. After confirming that each sample was at least 98% TMT labelled, total reporter ion intensities were used to normalise pooling of the remaining samples, such that the total peptide content between samples was as close to a 1:1 ratio as possible in the final pool. This pool was dried in a vacuum centrifuge to evaporate the majority of the acetonitrile. The sample was then acidified to a final 0.1% trifluoracetic acid (~200 µL volume) and formic acid was added until visible precipitation of the sodium deoxycholate was observed. Four volumes of ethyl acetate were then added and the sample vortexed vigorously for 30 s. The sample was then centrifuged at 15,000 g for 5 min at RT to effect phase separation. The lower (aqueous) phase was then withdrawn to a fresh microfuge tube using a gel loading pipette tip. If the event that any obvious sodium deoxycholate contamination was remaining, the two-phase extraction with ethyl acetate was repeated. The sample was partially dried in a vacuum centrifuge and brought up to a final volume of 1 mL with 0.1% trifluoracetic acid. Formic acid was added until the pH was <2, which was confirmed by spotting onto pH paper. The sample was cleaned up by solid phase extraction using a 50 mg tC18 SepPak cartridge (Waters) and a PPM. The cartridge was wetted with 1 mL 100% methanol followed by 1 mL acetonitrile, equilibrated with 1 mL 0.1% trifluoracetic acid and then the sample loaded slowly. The sample was passed over the cartridge twice. The cartridge was washed 3x with 1 mL 0.1% trifluoracetic acid before eluting sequentially with 250 µL 40% acetonitrile, 70% acetonitrile and 80% acetonitrile and then dried in a vacuum centrifuge.

### Basic pH reversed phase fractionation

TMT labelled samples were resuspended in 40 µL 200 mM ammonium formate pH10 and moved to a glass HPLC vial. BpH-RP fractionation was carried out on an Ultimate 3000 UHPLC system (Thermo Scientific) equipped with a 2.1 mm × 15 cm, 1.7 µm Kinetex EVO column (Phenomenex). Solvent A was 3% acetonitrile, solvent B was 100% acetonitrile, solvent C was 200 mM ammonium formate (pH 10). During the analysis, solvent C was maintained at a constant 10%. The flow rate was 500 µL/min and UV was monitored at 280 nm. Samples were loaded in 90% A for 10 min before a gradient elution of 0–10% B over 10 min (curve 3), 10–34% B over 21 min (curve 5), 34–50% B over 5 min (curve 5) followed by a 10 min wash with 90 % B. 15 s (100 µL) fractions were acquired throughout the run. Fractions that contained peptide (determined by A280) were then recombined across the gradient to preserve orthogonality with on-line low pH RP separation. For example, fractions 1, 25, 49, 73, 97 were combined and dried in a vacuum centrifuge and stored at -20°C until LC-MS analysis. In this manner, 24 fractions were generated.

### Mass spectrometry

Samples were analysed on an Orbitrap Fusion instrument on-line with an Ultimate 3000 RSLC nano UHPLC system (Thermo Fisher). Samples were resuspended in 10 µL 5% DMSO/1% trifluoracetic acid. 5 µL of each fraction was injected for TMT experiments. The trapping solvent was 0.1% trifluoracetic acid, analytical solvent A was 0.1% formic acid, and solvent B was acetonitrile with 0.1% formic acid. Samples were loaded onto a trapping column (300 µm × 5 mm PepMap cartridge trap (Thermo Fisher)) at 10 µL/min for 5 min. The samples were separated on a 75 cm × 75 µm i.d. 2 µm particle size PepMap C18 column (Thermo Fisher). The gradient was 3–10% B over 10 min, 10–35% B over 155 min, 35–45% B over 9 min followed by a wash at 95% B for 5 min and re-equilibration at 3% B. Eluted peptides were introduced to the MS by electrospray, by applying 2.1 kV to a stainless-steel emitter (5 cm × 30 µm (PepSep)). During the gradient elution, mass spectra were acquired using Tune v3.3 and Xcalibur v4.3 (Thermo Fisher).

### Data processing

Data were processed with PeaksX+, v10.5 (Bioinfor). One might alternatively use the open-source software MaxQuant to perform a similar analysis. Files in .raw format were searched iteratively in three rounds, with unmatched DeNovo spectra (at 0.1 % PSM FDR) from the previous search used as the input for the next. The three rounds were as follows 1) Swiss-Prot Human + common contaminants 2) The same databases as search 1, but allowing semi-specific cleavage 3) trEMBL Human, with specific cleavage rules. Identified proteins and their reporter ion intensities were imported to R (v4.0.3) and submitted to statistical analysis using LIMMA v3.15. LIMMA is a moderated t-test available through the Bioconductor package. LIMMA p-values were corrected for multiple hypothesis testing by the Benjamini-Hochberg method to generate an FDR (q-value) for each comparison. Data are made available via the Protein Interactions Database (PRIDE) ^23^ with the dataset identifier PXD034135 ^24^.

### Data analysis

Two separate measures of fold-change were calculated to identify cell-type specific proteins within the single replicate analysis of sorted hAECs. A maximum-fold change between any of the sorted TUBA+, NGFR+, CEACAM6+ and unstained samples was calculated for each protein. Fold-changes of each of the sorted populations compared to unsorted cells was also calculated. Proteins were selected for clustering analysis if they had a maximum fold-change > 2 between any of the sorted cell types and ensuring fold-change to unsorted cells was > 1, indicating an enrichment within a particular sorted population. A total of 1,738 proteins from 7,917 detected were classed as cell-type specific by this definition. TMT reporter ion intensities of sorted populations (TUBA+, NGFR+, CEACAM6+, unstained) were scaled and clustered into six groups using the kmeans function in R base stats package (v4.1.2).

Statistical analysis of Calu-3 datasets to detect host proteins changing between mock, bystander and infected cells following SARS-CoV-2 infection was performed independently for each timepoint. Proteins were filtered ensuring they were detected across all TMT reporter channels and with more than one unique peptide within the analysed timepoint. Statistical tests were performed with the aov and p.adjust functions in R base stats package to calculate Benjamini-Hochberg corrected p-values for changes in protein abundance across sorted mock, bystander and infected samples. Host proteins with a p-value <0.05 and a maximum fold-change between conditions of > 1.5-fold were selected for k-means clustering. TMT reporter ion intensities were scaled by the mean intensity of mock samples from the equivalent time-point. The mock-scaled means of each condition for all timepoints were utilised to cluster proteins into five groups using the kmeans function in R base stats package.

### Functional gene set enrichment analyses

Over-representation analysis (ORA) and Gene Set Enrichment Analysis (GSEA) of clustered proteins or up/down-regulated proteins on infection were performed using the clusterProfiler package (v4.2.1) in R with the comparecluster and enricher functions or GSEA function respectively ^25^. Gene sets utilised in ORAs were downloaded using the msigdbr R package (v7.4.1) and included “Hallmark”, “Reactome”, “KEGG” and Gene Ontology “Biological Process” and “Cell Component” categories ^26– 28^. The clusters defined in the hAEC cell-type proteomics were utilised as gene sets in GSEA of proteins up or downregulated upon infection of ciliated cells. The total detected proteome in each experiment was defined as the background to test for enrichment of gene sets.

## Results

### Proteomes of the major cells of the human airway epithelium

**Figure 1.**
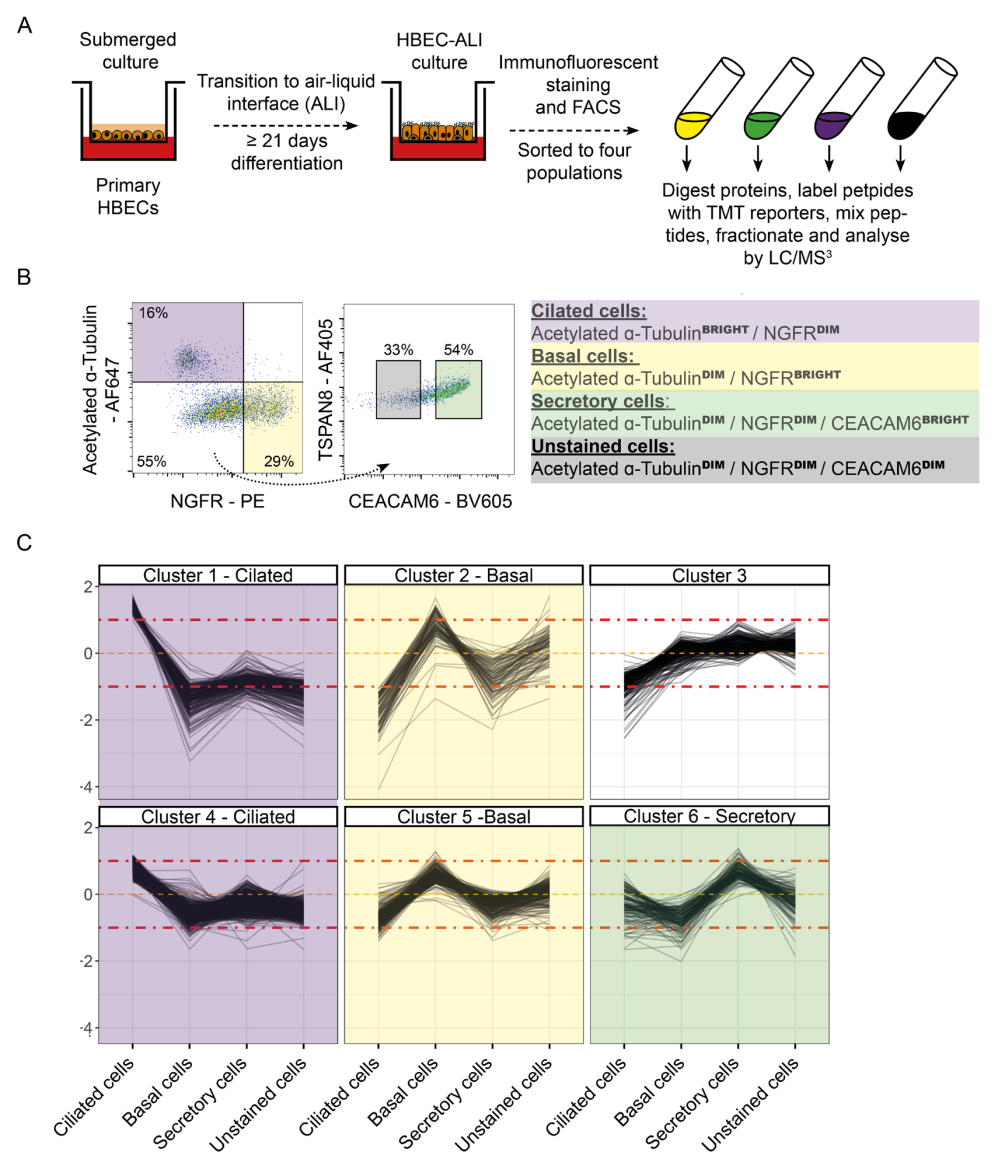
Proteomic characterisation of the key cell types of the pseudostratified epithelium of primary human airway epithelial cells (hAECs) differentiated at the air-liquid interface (ALI). ( **A**) Schematic of the hAEC-ALI experimental setup. ( **B**) Flow sorting strategy employed to discriminate the different cell types for characterisation. ( **C**) Expression profiles of proteins significantly enriched in each cell type. Proteins falling into cluster 3 were not enriched in any single cell type.

We initially wished to characterise the proteomes of the three predominant cell types of the pseudostratified epithelium. Primary normal bronchial epithelial cells were differentiated by culture on a transwell insert at the ALI for over three weeks. We chose a panel of antibodies previously shown to discriminate the major cell types of the pseudostratified epithelium ^29^ ciliated cells (acetylated TUBA+), basal cells (NGFR+) and secretory cells (CEACAM6+). Cells differentiated at the ALI were fixed, stained and sorted for these markers by FACS, to allow cell-type specific proteomic analysis ( Figure 1A, B), quantifying **7,918** proteins. Data from all proteomics experiments is available in an interactive spreadsheet format as **underlying data**, **Table S1** ^30^.

Proteins were clustered for their pattern of expression across the four sorted cell populations, to group together proteins that were only highly expressed in a single cell type ( Figure 1C). As expected, gene set over-representation analysis confirmed the predicted protein enrichment for each purified cell population. Ciliated cells were highly enriched for proteins which constitute cilia, along with proteins involved in cilium biogenesis, assembly and movement; basal cells were enriched for proteins which make up the hemidesmosome, as well as proteins involved in cell-cell and cell-ECM adhesion and DNA replication; secretory cells were enriched for proteins secreted into the extracellular space **(Underlying data**, **Figure S1A**) ^30^.

### Quantitative proteomics of SARS-CoV-2 infected primary ciliated cells

**Figure 2.**
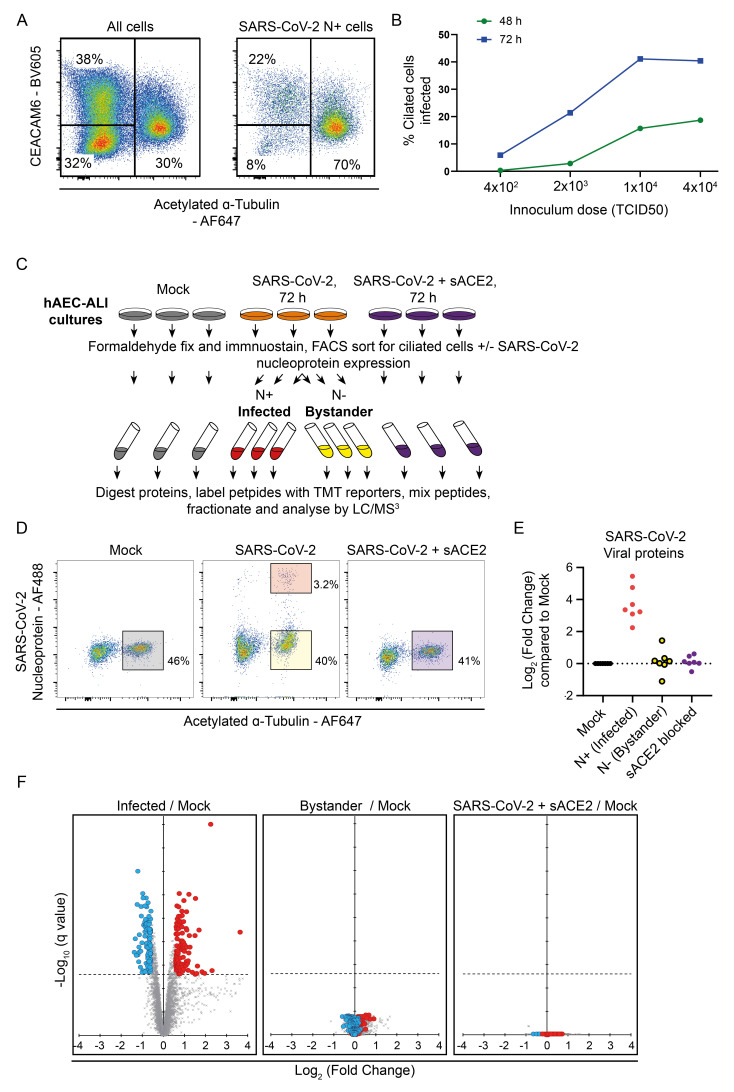
Quantitative proteomic analysis of SARS-CoV-2 infected hAEC-ALI ciliated cells. ( **A**) Example flow cytometry of SARS-CoV-2 infection of hAEC-ALI cells. Expression of CEACAM6 and acetylated alpha-tubulin in all cells of the epithelium (left), and in cells gated for SARS-CoV-2 nucleoprotein (right). ( **B**) Example time-course of infection of SARS-CoV-2 in hAEC-ALI cells. ( **C**) Schematic of the experimental design for proteomic analysis of SARS-CoV-2 infection of hAEC-ALI cells. ( **D**) Example flow cytometry from the proteomics experiment described in ( **C**). ( **E**) Quantification of SARS-CoV-2 proteins in this proteomics experiment. ( **F**) Scatterplots displaying pairwise comparisons between infected, bystander, sACE2-blocked and mock-infected cells. Each point on the scatterplot represents a human protein, plotted by the log2 (fold change) on the x-axis and the statistical significance of that change on the y-axis. q values were determined using LIMMA with Benjamini-Hochberg adjustment. Proteins significantly downregulated by more than 1.5-fold when infected cells are compared to the mock condition are highlighted in blue, proteins significantly upregulated by more than 1.5-fold are highlighted in red.

Our initial experiments indicated that the ciliated cell compartment is by far the most susceptible to SARS-CoV-2 infection, consistent with previous reports ^1– 3^. For example, Figure 2A shows a representative plot of SARS-CoV-2 infected hAEC-ALI cells stained for CEACAM6 and acetylated alpha-tubulin 72 h post infection. Figure 2A (left panel) shows all cells, with ciliated cells (acetylated tubulin high) representing 30% of the population. Figure 2A (right panel) shows the same sample after gating for SARS-CoV-2 positive cells, with ciliated cells now making up 70% of infected cells. Previous work modelling SARS-CoV-2 infection in hAEC-ALI has indicated that infection peaks between 48 h to 96 h post infection ^1, 2, 31, 32^, even where the virus inoculum dose used should be sufficient to infect all cells in the first round of infection, i.e. with an MOI >3 ^1, 32^. This was consistent with our preliminary experiments showing a substantial increase in infected cells at 72 h compared to 48 h, even where the initial virus dose appears to be saturating ( Figure 2B). In the same system, we have demonstrated that the addition of camostat mesylate 24 h & 48 h after viral inoculation leads to a greatly reduced number of infected cells at 72 h ^33^. This suggests that the increasing number of infected cells up to 72 h represents spread of the virus into cells that were unable to be infected by the initial inoculum (but could be infected in subsequent rounds of viral release).

As we wanted to sort for a pure population of infected cells for our proteomics analysis, in order to remove confounding effects of uninfected bystander cells, we examined only ciliated cells. Infected basal and secretory cells were relatively rare, providing insufficient material for independent proteomic analysis, and if we sorted only for nucleoprotein expression without selecting a single cell type, the infected and uninfected populations would consist of greatly different populations. Mature hAEC-ALI cells were infected with SARS-CoV-2 and harvested and fixed at 72 hpi. Ciliated cells were identified by the detection of acetylated alpha-tubulin and were sorted into sub-populations of SARS-CoV-2 nucleoprotein positive (infected) and negative (bystander) populations ( Figure 2C, D). Uninfected ciliated cells, and ciliated cells exposed to SARS-CoV-2 in the presence of soluble ACE2 (sACE2) to block infection were also analysed as a control for the effect of the virus preparation.

A total of **5,709** host protein accessions were quantified, in addition to the SARS-CoV-2 proteins Rep1AB polyprotein, nucleoprotein, spike, membrane, ORF3A, ORF9B, and ORF7A. As expected, SARS-CoV-2 proteins were highly expressed only in the infected cells ( Figure 2E). When considering host proteins, a comparison of infected cells with the uninfected (mock) control cells ( Figure 2F, left panel) revealed 530 significant (q < 0.05) changes in infected cells. Of these **226** proteins changed by more than 1.5-fold, with **109** depleted, and **117** proteins increased in abundance. These proteins were altered as a direct consequence of viral infection, as comparisons of the uninfected ‘bystander’ cells, or cells exposed to SARS-CoV-2 in the presence of sACE2, to the mock population ( Figure 2F, centre and right proteins) identified no proteins showing significant changes in abundance.

Individual downregulated proteins previously implicated in SARS-CoV-2 infection include Syndecan-4 (SDC4) (53% reduction, q = 0.02), which (along with other heparan sulfated proteins) has been implicated in facilitating SARS-CoV-2 entry via an interaction with SARS-Cov-2 spike ^34– 37^. We also found a significant reduction of the anti-viral signalling protein MAVS (depleted by 39%, q = 0.01), consistent with reports that MAVS is targeted for proteasomal and/or mitophagy-mediated degradation by SARS-CoV-2 proteins ^38, 39^.

**Figure 3.**
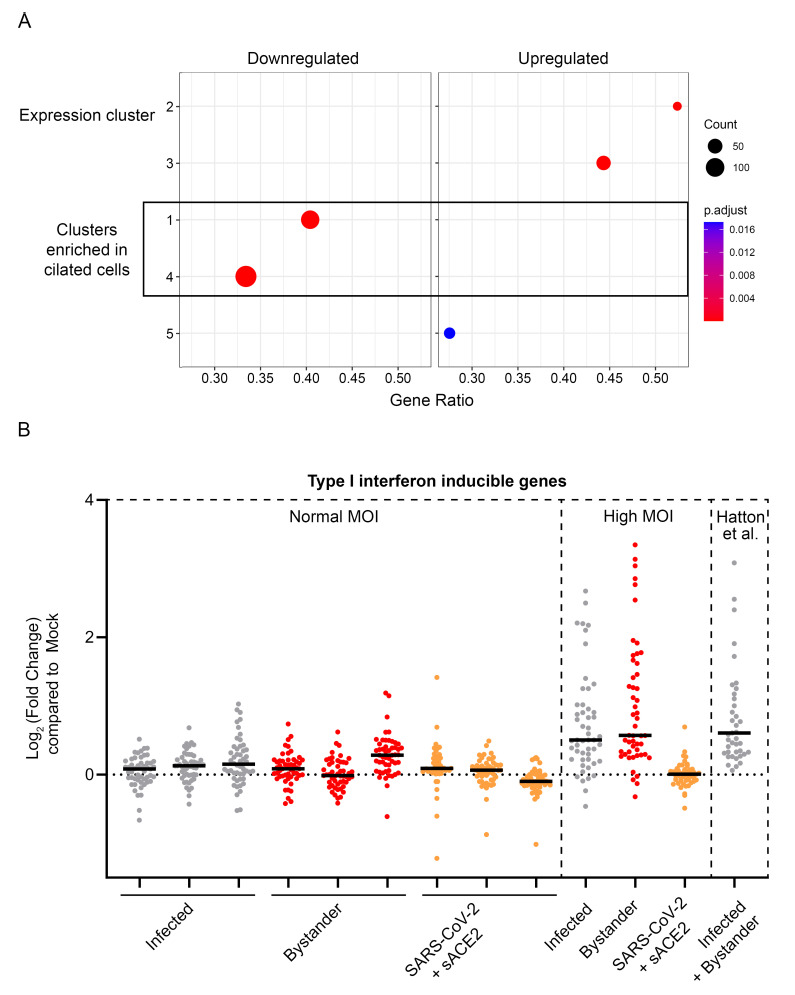
Analysis of proteins regulated by SARS-CoV-2 in hAEC-ALI cells. ( **A**) Proteins down or upregulated in SARS-CoV-2 infected hAEC-ALI cells (highlighted in blue or red in Figure 2F), were tested for enrichment in the clusters of proteins which are most characteristic for each cell type (as shown in Figure 1C). ( **B**) Behaviour of a list of type I interferon induced genes in the SARS-CoV-2 infection of hAEC-ALI ciliated cells, or the same cells infected at a higher MOI, or the same proteins as quantified in the SARS-CoV-2 infection of hAEC-ALI cells by Hatton *et al*. The y-axis shows the fold change when the condition shown on the x-axis is compared to the mock condition from the same experiment. As the analysis in Hatton *et al*., does not include a sorting step, the analysed well contains both infected and bystander populations.

Notably, the downregulated proteins were enriched for proteins characteristic of ciliated cells ( Figure 3A). This loss of proteins which define features of ciliated cells is consistent with the de-differentiation of the ciliated cell phenotype previously reported in SARS-CoV-2 infection ^40^.

Similar approaches to map proteomic changes to air-liquid interface differentiated human primary epithelial cells have been reported. Hatton *et al.* ^41^ analysed differentiated primary epithelial cells from six donors infected with SARS-CoV-2 at 72 hpi, comparing all cells in the infected well with uninfected cells from separate wells. As expected with the different methodologies employed, levels of concordance between the two datasets were relatively low ( **Underlying data**, **Figure S1B**) ^30^, with no correlation in upregulated proteins, but some overlap in depleted proteins. We have highlighted those proteins depleted in both datasets, such as SDC4 ( **Underlying data**, **Figure S1C**) ^30^.

There is one obvious cause of discrepancy between the data we report here and that reported by Hatton *et al*., and some of the transcriptional approaches in primary differentiated epithelial cells. In Hatton *et al*., upregulated proteins are generally dominated by type I interferon inducible genes. By contrast, we observed little or no evidence of a type I interferon response in either infected or bystander cells ( Figure 3B).

Our primary differentiated epithelial cells are fully capable of generating a type I interferon response to SARS-CoV-2. We conducted a pilot proteomics experiment of cells plated on a larger size transwell insert at a higher MOI (approximately 7.5≥ higher than in the previous experiments). A robust type I interferon response was measured in both the infected and bystander cells ( Figure 3B) comparable with the type I interferon response reported by Hatton *et al*.

### Quantitative proteomics of SARS-CoV-2 in a lung epithelial cell line, Calu-3

**Figure 4.**
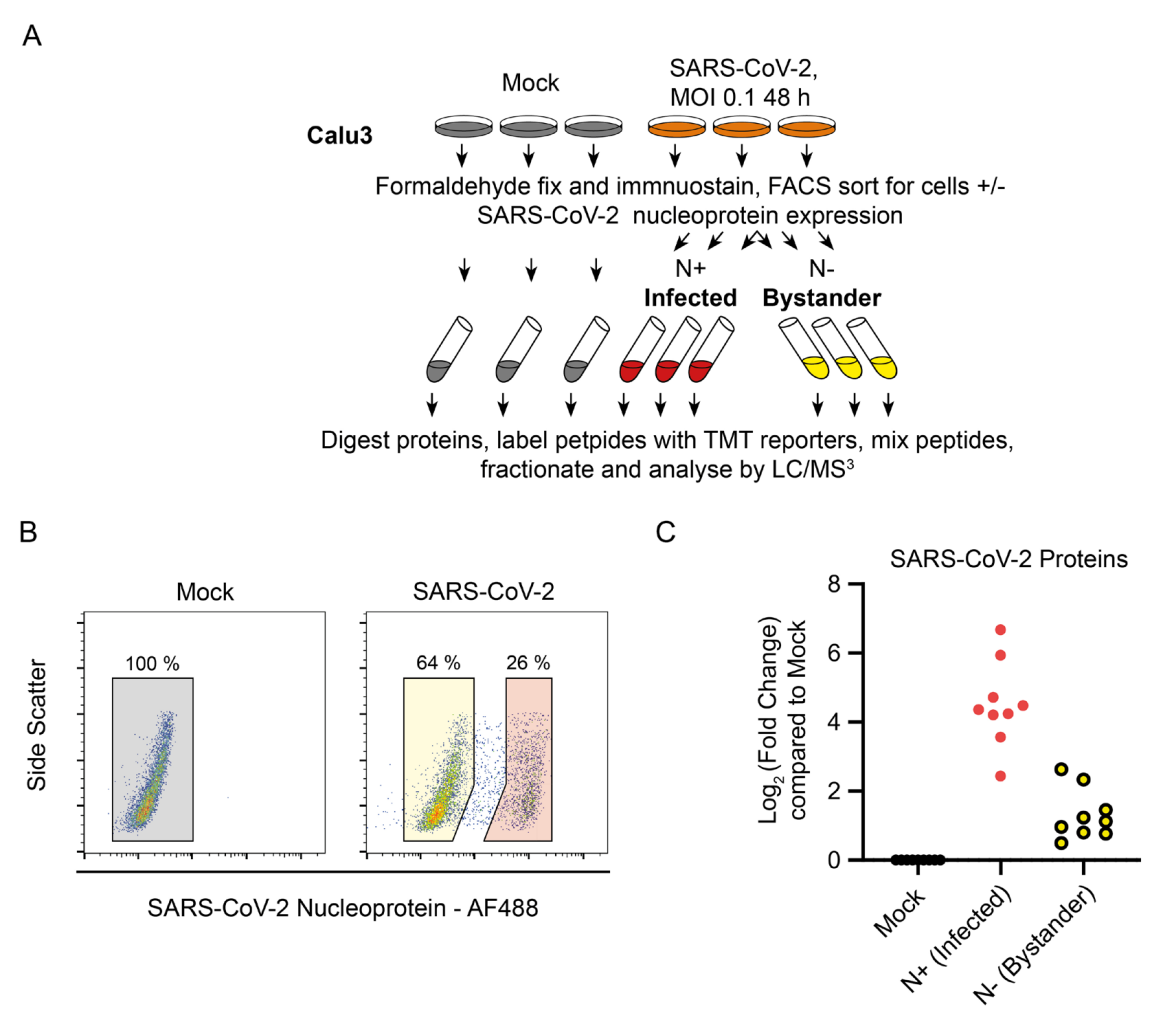
Outline of a single time-point SARS-CoV-2 proteomics experiment. ( **A**) Schematic of the experimental design for proteomic analysis of SARS-CoV-2 infection of Calu-3 cells. ( **B**) Example flow cytometry from the proteomics experiment described in ( **A**). ( **C**) Quantification of SARS-CoV-2 proteins in this proteomics experiment.

While primary airway epithelial cells differentiated at the ALI represent one of the most compelling models for the human upper and lower airway epithelium, this model presents a number of technical challenges. Most SARS-CoV-2 research therefore also use cell lines such as Calu-3, which was originally derived from a male lung adenocarcinoma. Calu-3 are one of the few airway epithelial cell lines which can be infected with SARS-CoV-2 without the need to express exogenous ACE2 or other host factors to facilitate infection. We therefore extended our proteomic analysis to SARS-CoV-2 infected Calu-3 cells. As with the hAEC-ALI experiments, we used flow cytometry to separate pure populations of N+ and N- cells from the same infected well ( Figure 4A), 48 h post infection. At this time point, 26% of the cells were infected, and after sorting, SARS-CoV-2 viral proteins were highly expressed in N+ infected cells, with some detection in the N- bystander cells ( Figure 4C). In this and subsequent experiments with Calu-3 cells, between 30–75% of cells, could not be infected with SARS-CoV-2 (e.g. the 25% infection attained in Figure 4B).

**Figure 5.**
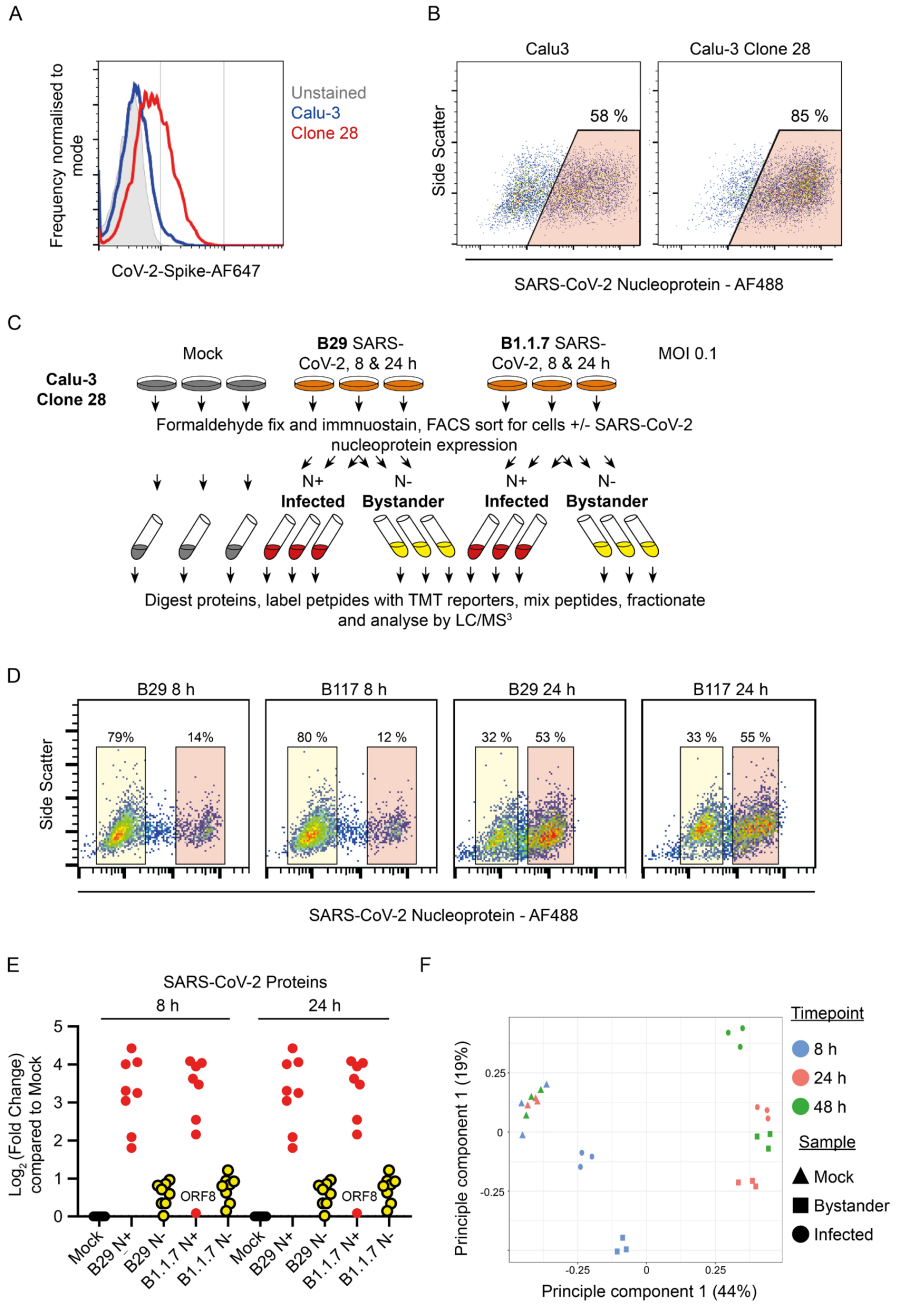
Outline of a multiple time-point SARS-CoV-2 proteomics experiment in an ACE-2 high Calu-3 clone. ( **A**) Staining for ACE2 using SARS-CoV-2 spike protein on Calu-3 cells and a Calu-3 clone, clone 28, identified with high ACE2 expression. ( **B**) Example SARS-CoV-2 infection and nucleoprotein staining 24 h after infection of Calu-3 cells or Calu-3 clone 28. ( **C**) Schematic of the experimental design for proteomic analysis of SARS-CoV-2 infection of Calu-3 cells. ( **D**) Example flow cytometry from the proteomics experiment described in ( **C**). ( **E**) Quantification of SARS-CoV-2 proteins in this proteomics experiment. ( **F**) Principal component analysis of the conditions included in the Calu-3 proteomics experiments described in Figure 4 and Figure 5.

To enhance SARS-CoV-2 infection, we single-cell cloned Calu-3 cells, screening for high ACE2 expression by flow cytometry. We identified a high-ACE2 expressing clone (clone 28) Figure 5A, which dramatically reduced the population of cells refractory to infection ( Figure 5B). In a second proteomics experiment this clone was infected with either B.29 or B.1.1.7 (Alpha) variant SARS-CoV-2 over a time-course of infection with cells sampled at 8 and 24 hpi ( Figure 5C). Example flow cytometry of one replicate of each infection condition is shown in Figure 5D. Again, viral proteins were greatly increased in the cells enriched for nucleoprotein ( Figure 5E). 

**Figure 6.**
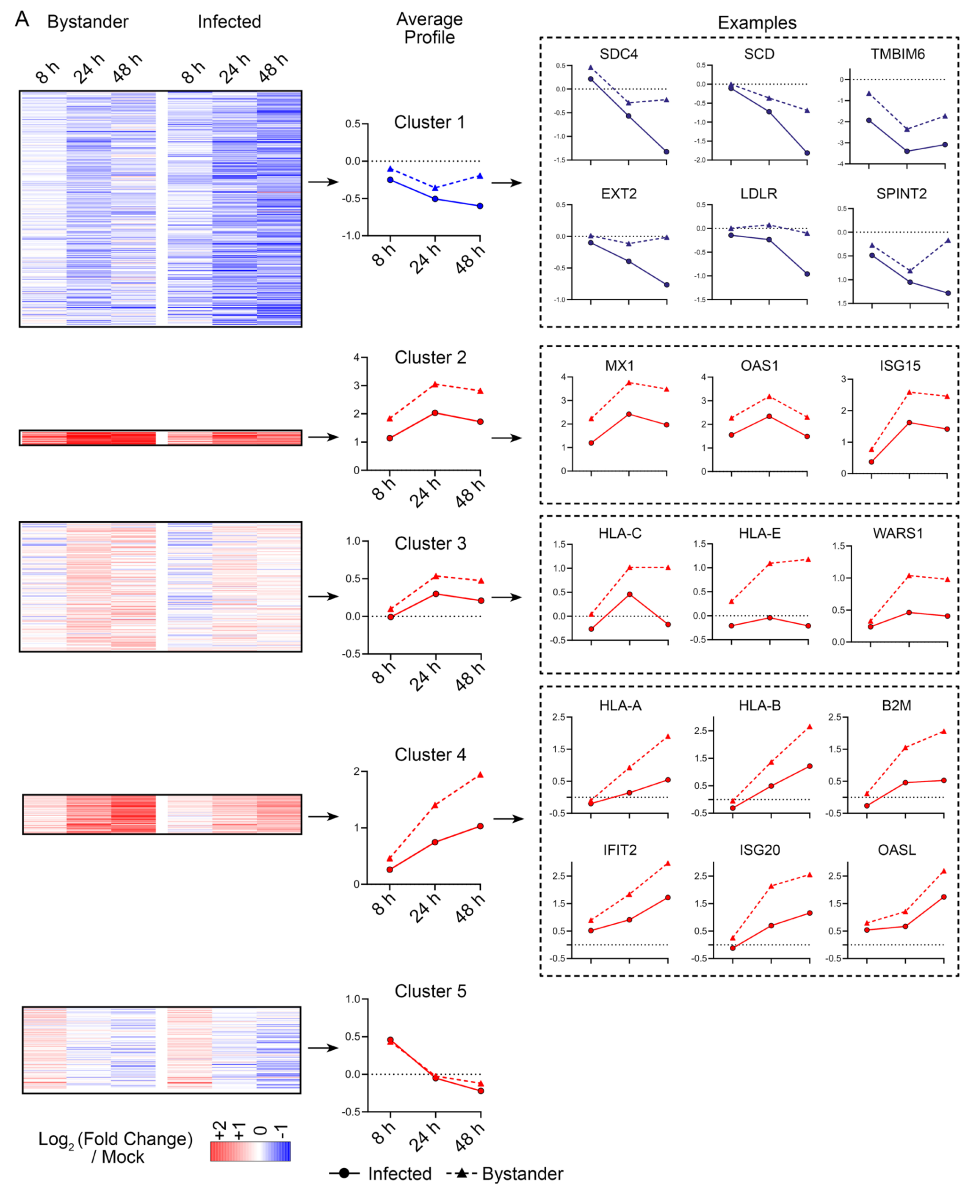
Analysis of proteins altered by SARS-CoV-2 infection of Calu-3 cells. ( **A**) Left, Heatmap showing the regulation of five clusters of proteins in bystander or infected cells at 8, 24, or 48 h. Data is combined from the two experiments described in Figure 4 and Figure 5. Centre, average time-course profiles of proteins within each cluster. Right, example proteins regulated within each cluster.

A principal component analysis (PCA) of the two proteomics experiments conducted in Calu-3 cells indicated that each triplicate was similarly clustered. Furthermore, as the infected and bystander cells from either 24 or 48 h post infection showed a similar clustering pattern ( Figure 5F), we analysed data from both experiments together as a single time course. A total of **6,844** human protein accessions were quantified across all three timepoints with more than one unique peptide detected in at least one timepoint. We identified **645** proteins as showing statistically significant changes between “Mock”, “Bystander” or “Infected” samples at any one of the three timepoints, with the criteria of (i) detected with >1 unique peptide (ii) a change in abundance of >1.5-fold and (iii) a Benjamini-Hochberg corrected ANOVA p-value <0.05 within the individual timepoint. Proteins were normalised to the timepoint specific “Mock” and subject to k-means clustering, with differentially regulated proteins grouped into five clusters based on their behaviour in infected and bystander cells across the time-course ( Figure 6A). These clusters were subject to gene set over-representation analysis ( **Underlying data**, **Figure S2A**) and had the following characteristics:

Cluster 1 represents proteins progressively downregulated in SARS-CoV-2 infection and is significantly enriched for proteins involved in several gene sets. These include heparan sulfated proteins such as **SDC4** and proteins related to the metabolism of heparan sulfate such as **EXT2**. Other gene sets enriched included proteins involved in cholesterol biosynthesis such as **SCD** and **RNF145**. Also depleted were the regulator of apoptosis TMBIM6 and the regulator of TMPRSS2 expression, SPINT2, both previously identified as being downregulated in transcriptional and proteomics datasets generated in SARS-CoV-2 infected cell lines and from infected patient cells ^42, 43^.

Clusters, 2, 3 and 4, represent proteins upregulated in SARS-CoV-2 infection, and were highly enriched for type I interferon inducible proteins ( Figure 6A). Importantly, type one interferon inducible proteins were consistently more upregulated in ‘bystander’ cells than in SARS-CoV-2 infected cells. To further demonstrate this effect, we compared the upregulation of a list of type I interferon inducible genes in infected and bystander cells, showing that these proteins were consistently more strongly upregulated in bystander cells ( **Underlying data**, **Figure S2B**). Cluster 5 contains proteins with a mixed pattern of regulation across the time-course and was not significantly enriched for proteins in any gene set tested.

**Figure 7.**
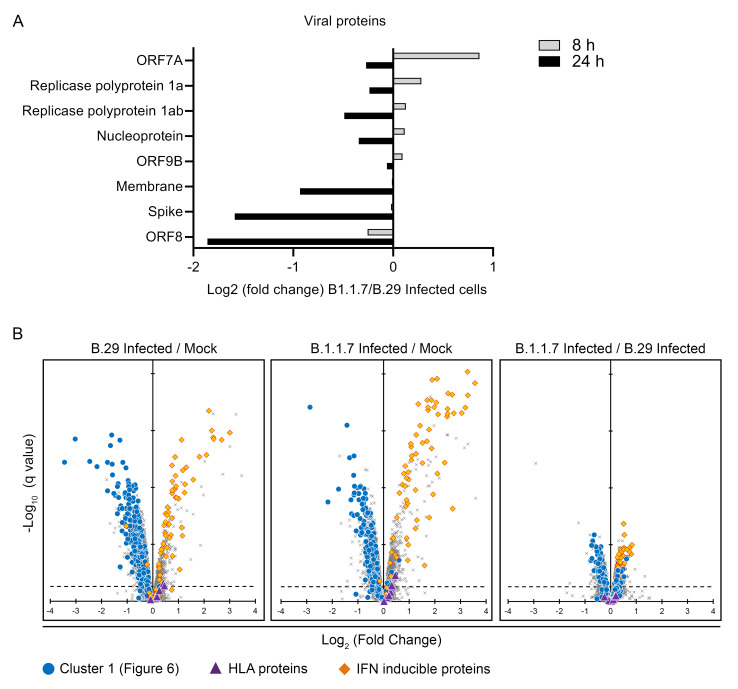
Comparison of B.29 and B.1.1.7 (Alpha) infection. ( **A**) Comparison of SARS-CoV-2 viral protein abundance between cells infected with SARS-CoV-2 B.29 and B.1.1.7. ( **B**) Scatterplots displaying pairwise comparisons between B.29 and B.1.1.7 infected cells and mock infected cells. Each point on the scatterplot represents a human protein, plotted by the log2 (fold change) on the x-axis and the statistical significance of that change on the y-axis. q values were determined using Limma with Benjamini-Hochberg adjustment. Proteins falling into the ‘cluster 1’ pattern of regulation in Figure 6A are highlighted in blue. IFN inducible genes are highlighted in red. MHC class I proteins are highlighted as purple triangles.

Finally, we compared the effects of the ‘first wave’ SARS-CoV-2 B.29 virus with B.1.1.7 (VOC Alpha). A previous study reported that several SARS-CoV-2 proteins involved in SARS-CoV-2 immune evasion were more strongly expressed by the alpha variant than ‘first wave’ variants, and as a result, alpha showed a much greater ability to suppress the type I interferon response ^18^. We found little difference in the expression of SARS-CoV-2 viral proteins when N+ sorted cells were compared at 8 or 24 h ( Figure 7A). The level of type I interferon response induced was also similar ( Figure 7B). There were no obvious differences in the behaviour of other proteins changed by SARS-CoV-2 infection between the two variants. Finally, we note that SARS-CoV-2 ORF8 has been proposed to deplete MHC class I proteins ^44^. As the alpha variant is deficient for ORF8, we determined whether MHC class I protein expression during infection differs compared with the B.29 virus. In cells infected with both variants, MHC class I proteins were slightly upregulated, consistent with the identifiable, but muted, induction of type I interferon in infected cells.

## Discussion

We present here a quantitative proteomic analysis of SARS-CoV-2 infection of two commonly used model systems, primary human airway epithelial cells differentiated at the air liquid interface (hAEC-ALI), and the lung epithelial cell line Calu-3. The findings in the two models were somewhat different with the major discordance being in the nature of the type I interferon response.

The timing and intensity of the type I IFN response in SARS-CoV-2 infection of ALI-hAEC models is the subject of varying reports, being described as either robust, impaired, delayed, or absent in previous studies using primary hAEC-ALI cells ^3– 5, 7, 45^. Here, we found that at 72 h post infection, there was very little evidence of a type I interferon response in infected or bystander ciliated cells, but a response was readily elicited at a higher dose of virus inoculum. Discrepancies between studies regarding the magnitude of the type I interferon response elicited in this model system may therefore depend on the specific experimental design.

Multiple innate immune evasion strategies of SARS-CoV-2 have now been described ^39, 46– 51^. The success of these mechanism seems to be at least in part responsible for severe disease as muted IFN responses in the airway early in infection correlated with severe disease ^52^. A failure to mount a prompt innate response may also contribute to the extended pre-symptomatic or asymptomatic period of infection which has facilitated the spread of SARS-CoV-2.

In the hAEC-ALI model used here, it appears that these immune evasion mechanisms are sufficient to prevent or delay a type I interferon response through three days of infection. In the Calu-3 model, the response is somewhat different, as a type I interferon response is induced, however, this response is significantly muted in the infected cells compared to uninfected bystander cells. Here our use of pure populations of infected cells was informative, as we were able to deconvolute this differential response, while a proteomic or transcriptomic analysis carried out on the entire population would only reveal a robust interferon response originating disproportionately from bystander cells.

We also observe distinct classes of proteins downregulated in ciliated hAEC-ALI and Calu-3 cells upon infection. Our cell-type resolved proteome of hAEC-ALI cells indicates a basis for this divergence. Ciliated cells within hAEC-ALI cell culture systems are the most distinct cell type in comparison to basal and secretory cells in terms of proteins expressed. These proteins which define ciliated cells are also enriched within the limited set of proteins decreased in abundance on SARS-CoV-2 infection, a finding supported by prior studies demonstrating a de-differentiation of ciliated cells upon infection ^40^. The difference in classes of down-regulated proteins between both models is therefore unsurprising, as Calu-3 cells lack many of the specialised proteins expressed in primary ciliated cells. However, the greater depth of the data acquired in Calu-3 cells allows insights not possible from the hAEC-ALI model. For example, identifying a decrease in abundance of multiple proteins involved in glycosaminoglycan and cholesterol biosynthesis, including the master transcriptional regulator SREBF2, both pathways associated with the biosynthesis of components implicated in SARS-CoV-2 entry ^36, 37, 53– 56^.

## Conclusions

In this work we have developed proteomic methodologies for the analysis of fixed, permeabilised and immunostained cells. This has allowed us to analyse the cell-type specific proteomes of hAEC-ALI cultures as well as pure populations of SARS-CoV-2 infected primary-derived ciliated cells and Calu-3 cell lines. These approaches have allowed us to demonstrate suppression of the interferon response in cells actively infected with SARS-CoV-2, and identified multiple candidate proteins and biological pathways which are affected by SARS-CoV-2 infection as a resource for future SARS-CoV-2 research.

## Data availability

### Underlying data

Figshare: Underlying data for ‘Quantitative proteomic analysis of SARS-CoV-2 infection of primary human airway ciliated cells and lung epithelial cells demonstrates the effectiveness of SARS-CoV-2 innate immune evasion’. https://doi.org/10.6084/m9.figshare.c.6029810.v2 ^30^

This project contains the following underlying data:

- Figure 1. Proteomic characterisation of the key cell types of the pseudostratified epithelium of primary human airway epithelial cells (hAECs) differentiated at the air-liquid interface (ALI). Data underlying this figure can be found in **underlying data Table S1** and **Table S2.**
- Figure 2. Quantitative proteomic analysis of SARS-CoV-2 infected hAEC-ALI ciliated cells. Data underlying this figure can be found in **underlying data Table S1** and **Table S3.**
- Figure 3. Analysis of proteins regulated by SARS-CoV-2 in hAEC-ALI cells. Data underlying this figure can be found in **underlying data Table S1** and **Table S3**
- Figure 4. Outline of a single time-point SARS-CoV-2 proteomics experiment. Data underlying this figure can be found in **underlying data Table S1** and **Table S4**
- Figure 5. Outline of a multiple time-point SARS-CoV-2 proteomics experiment in an ACE-2 high Calu-3 clone. Data underlying this figure can be found in **underlying data Table S1, Table S5** and **Table S6**
- Figure 6. Analysis of proteins altered by SARS-CoV-2 infection of Calu-3 cells. Data underlying this figure can be found in **underlying data Table S1, Table S5** and **Table S6**
- Figure 7. Comparison of B.29 and B.1.1.7 (Alpha) infection. Data underlying this figure can be found in **underlying data Table S1, Table S5** and **Table S6**
- **Figure S1.** **(A)** Enrichment of proteins characteristic for the different cell types of the hAEC-ALI epithelium for biological pathways defined by ‘Reactome’. **(B)** Upper, proteins which are unchanged (left), depleted (centre) or upregulated (right) in SARS-CoV-2 infected hAEC-ALI cells in this report (i.e. proteins shown in grey, blue or red respectively in Figure 2D, with regulated proteins having a q-value of <0.05 and a fold change of >1.5 fold) are divided by their behaviour in SARS-CoV-2 infection of hAEC-ALI cells in Hatton *et al*. Proteins are categorised in the pie chart as up or down-regulated in Hatton *et al*. if they are significantly changed in either direction (with no fold change cut-off). Lower, proteins which are unchanged (left), depleted (centre, q<0.05, fold change >1.5 fold) or upregulated (right, q<0.05, fold change >1.5 fold) in Hatton *et al*. infection of hAEC-ALI cells characterised by their behaviour in SARS-CoV-2 hAEC-ALI cells in this report. **(C)** Proteins significantly depleted in SARS-CoV-2 infection of hAEC-ALI cells in this report and in Hatton *et al*.
- **Figure S2.** **(A)** Enrichment of proteins in each of the temporal clusters defined in Figure 6 for involvement biological pathways defined by ‘Hallmark’ and ‘Reactome’. **(B)** Behaviour of a list of type I interferon inducible genes in the three time-points of infection in bystander or infected cells compared to mock, uninfected cells.
- **Table S1.** Interactive.xlsx containing all processed datasets.
- **Table S2.** Proteomic characterisation of the key cell types of the pseudostratified epithelium of primary human airway epithelial cells (hAECs)
- **Table S3.** Quantitative proteomic analysis of SARS-CoV-2 infected hAEC-ALI ciliated cells
- **Table S4.** Single time-point (48 h) SARS-CoV-2 proteomics experiment
- **Table S5.** 8 h timepoint of a multiple time-point SARS-CoV-2 proteomics experiment in an ACE-2 high Calu-3 clone
- **Table S6.** 24 h timepoint of a multiple time-point SARS-CoV-2 proteomics experiment in an ACE-2 high Calu-3 clone
- Data are available under the terms of the Creative Commons Attribution 4.0 International license (CC-BY 4.0)

### Accession numbers

PRIDE Project: Proteomics mass spectrometry data for *Homo sapiens* ^24^. Accession number PXD034135; https://identifiers.org/pride.project:PXD034135
