# Supplementary material for: Quantitative proteomic analysis of SARS-CoV-2 infection of primary human airway ciliated cells and lung epithelial cells demonstrates the effectiveness of SARS-CoV-2 innate immune evasion
Source: Wellcome Open Res. 2022 Sep 7;7:224. [Version 1] doi: 10.12688/wellcomeopenres.17946.1 (PMC9706147; doi:10.12688/wellcomeopenres.17946.1)
Supplement: Peer review history

# Peer review: Reviewer response for version 1

**DOI:** 10.21956/wellcomeopenres.19888.r53327

**Published:** 2022-11-28

**Authors:** Fabian Schmidt

**Affiliations:** 1 Laboratory for Applied Virology and Precision Medicine, King Abdullah University of Science and Technology (KAUST), Thuwal, Saudi Arabia

In this work, Crozier, Greenwood, Williamson, et al. have developed methodologies to document the proteome in specific cell types found in the pseudostratified epithelium of primary human airway epithelial cells differentiated at the air-liquid interface. They then apply their strategy to dissect the timing and intensity of the type I IFN response in this model system in response to SARS-CoV-2 infection. They complement their findings with data acquired in the human epithelial cell line Calu-3, enabling them to investigate the proteomic changes at greater depth.

 The rationale and results are set convincingly and exhaustively in context with existing literature. Indeed, the addressed topic remains a subject of varying reports, and the pathways and mechanisms exploited by SARS-CoV-2 in the host cells remain highly relevant in SARS-CoV-2 research.

 Their approach has allowed the authors to demonstrate suppression of the interferon response in cells actively infected with SARS-CoV-2 and bystander cells. Their findings identified and confirmed multiple candidate proteins and biological pathways affected by SARS-CoV-2 infection. 

 This article convinces through the experimental design, e.g., a single cell cloning step to enrich for ACE2 expression on Calu-3 cells and, in general, well-documented workflows in the method sections. Furthermore, the author's approaches are made easily accessible by schematics in the figures that complement the results. In conclusion, the innovative methodology, as well as the quality and accessibility of the presented data, will place this manuscript as a trustworthy resource for future SARS-CoV-2 research.

Is the work clearly and accurately presented and does it cite the current literature?

Yes

If applicable, is the statistical analysis and its interpretation appropriate?

Yes

Are all the source data underlying the results available to ensure full reproducibility?

Yes

Is the study design appropriate and is the work technically sound?

Yes

Are the conclusions drawn adequately supported by the results?

Yes

Are sufficient details of methods and analysis provided to allow replication by others?

Yes

Reviewer Expertise:

Previously: SARS-CoV2 pseudotype assays, humoral immune response to SARS-CoV2, immune evasion by spike mutations. Currently: Synthetic virology and gene therapy

I confirm that I have read this submission and believe that I have an appropriate level of expertise to confirm that it is of an acceptable scientific standard.

# Peer review: Reviewer response for version 1

**DOI:** 10.21956/wellcomeopenres.19888.r52705

**Published:** 2022-11-28

**Authors:** Áine McKnight

**Affiliations:** 1 Queen Mary University of London, Faculty of Medicine and Dentistry, Blizard Institute, London, UK

The paper by Crozier *et al.* reports on a proteomic evaluation of cells infected with the SARS-CoV-2 ancestral (or ‘first wave’) and Alpha variants. The target cells were primary human airway epithelia after differentiation in an air-liquid culture system but they also compared with a lung epithelial cell line Calu-3 infected cell clone.  They find that the two models are different with respect to the type 1 IFN response.

 The authors should add statistical p- values to most of their figures. This is the only issue that needs to be addressed. The following comments are points for discussion, debate or advisory. The paper stands on its own as a good scientific, rigorous paper. 

 Differentiation in an air-liquid culture system results in a mixed population of basal, secretory and ciliated cells. Because the ciliated cells were the most abundant and most susceptible to SARS-CoV-2 infection they purified populations by flow cytometric sorting followed by specific proteomic analysis. The cell specific segregation of individual marker proteins, determined by mass spectrometry, attested to the success of this approach. In the separated ciliated population they identified 109 proteins that increased after infection and 117 that decreased more than 1.5 fold including Syndecam-4 and MAVS previously implicated in SARS-CoV-2 infection. Interestingly, but not surprisingly, proteins associated with ‘ciliation’ were down modulated which concurs with reports that SARS-CoV-2 infection de-differentiation.

 The results differ notably from a similar screen by Hatton *et al. *There is no overlap in up regulated proteins and very little with those that are down modulated. As the authors point out the methodologies differ slightly- Hatton *et al* compared infected with uninfected cells in separate wells. 

 One noted possible cause for discrepancy put forward was that Hatton *et al’*s experiment induced a type 1 IFN response and these response proteins dominated.  This appears a likely explanation as here it is later shown (Fig 6A) that ‘bystander’ cells present in the mixed population (in Hatton’s experiments) would have induced ISGs. Nonetheless because of this difference here they checked that the cells in their system were capable of type 1 IFN induction. Thus they challenged cells with a higher MOI of over 7x more than the initial experiment and saw a robust type 1 IFN response in infected and in bystander cells. But were these gene sets overlapping with those of Hatton et al?  Did Hutton *et al* use a similar MOI? In the experiments described here with the ‘lower’ MOI experiment would a longer incubation with virus and more rounds of infection induce a similar IFN response?

 Are the SARS-CoV-2 variants used in Hatton *et al* the same as those in this study? Actually which SARS-CoV-2 variant was used in these initial experiments? It would be useful to explain that the lineage B.29 is the Ancestral-Wuhan Hu-1 strain earlier in the manuscript and whether it has the D164G mutation.

 Next, this study compares proteomic changes in infected and uninfected ‘bystander’ Calu-3 (cloned calu-3.28?) lung epithelial cells.  Proteins were normalised to mock infected and grouped into five clusters. Cluster 1 consisted of proteins which were progressively down modulated in virus infection and more so in the infected cells compared to the ‘bystander’ cells. Some of these have been previously reported to be affected during infection. Clusters 2, 3 and 4 were proteins up regulated in infections and were highly enriched for type 1 IFN inducible proteins. Importantly the bystander cells expressed more of these proteins. A nice result which I think deserves more discussion. Can you clarify exactly what bystander cells are? What exactly are the proteins up-regulated in these cells in fig 4C? I would suggest that these cells experience an ‘aborted’ rather than they were totally refractory to infection. This would support why there was not much overlap with Hatton *et al study*. In separating out the two cell populations the aborted infection was enriched for cells that successfully halted infection with a potent IFN response. I think the authors are eluding to this idea but its not coming across.

 They also compare infection between B.29 and B.1.1.7 (Alpha) variants and fail to reproduce the observations by Thorne *et al* (18) that the B.1.1.7 variant showed a much greater ability to suppress type 1 IFN response. I think it might be worth expanding on this discrepancy. It’s not clear which cells were used the experiment here but I assume it was the calu-3.28 clone.  Ansari *et al* ( https://doi.org/10.12688/wellcomeopenres.16559.1) show that there is a negative correlation between ISG expression and ACE2 expression.  If the Calu-3.28 was cloned for high ACE2  expression could this not mean low ISG expression? Could this have a role in the difference? Also could the ‘bystander cells’ be expressing less ACE2 and therefore more IFN responsive.

 Is there a difference between the ‘first wave’ isolates used for comparison both studies? Was the viral input in both studies the same? How was viral input determined?  Variants might have a different degree of cytopathic effect in cells. If CPE is used its possible that one virus may have more or less CPE per infectious unit which may be different in different cell lines (Huh7 vs Calu-3 vs Calu3.28).  None of this takes from the worth of the data but I think useful to consider.

 The work adds greatly to our understanding of the relationship between SARS-CoV-2 and the interaction of the host response.  The methodology developed will be very helpful in the field at elucidation virus-host interactions. They represent a rich source of unique data. They also lend caution to the interpretation of results and the comparison between different culture systems and virus variants. This will be a rich resource for SARS-CoV-2 research. Overall the work is clearly presented and the literature appropriately cited. The experiments are well designed, and data analysis nicely presented and is robust. The conclusions drawn in the discussion are broadly appropriate.

 However, I don’t agree that one can conclude that they have demonstrated that SARS-CoV-2 suppresses the IFN response after infection. It could well be the case but this premise is still open to counter argument. I would suggest that the cells that were successfully infected in the mixed cultures (N+) were more susceptible *because *these host cells were less capable of inducing a rapid IFN response. The ‘bystander’ cells (N-) then represent abortive infections of cells that responded rapidly, mounting rapid and potent expression of ISGs. It would be interesting to see how far into the replication cycle the virus is successful in such cells. This could be tested for RdRP protein expression or qPCR of sub-genomic RNAs. Also might all the basal and secretory cells in the mixed cultures be considered to be abortive infections? It would be valuable to hear any thoughts the authors have on why this might be.

 By the way can you say what camostat mesylate is or does to infection?

Is the work clearly and accurately presented and does it cite the current literature?

Yes

If applicable, is the statistical analysis and its interpretation appropriate?

Yes

Are all the source data underlying the results available to ensure full reproducibility?

Yes

Is the study design appropriate and is the work technically sound?

Yes

Are the conclusions drawn adequately supported by the results?

Yes

Are sufficient details of methods and analysis provided to allow replication by others?

Yes

Reviewer Expertise:

Virus host interactions

I confirm that I have read this submission and believe that I have an appropriate level of expertise to confirm that it is of an acceptable scientific standard.
